# COVID-19-associated pulmonary aspergillosis in intensive care unit: A real-life experience

**DOI:** 10.1016/j.heliyon.2024.e24298

**Published:** 2024-01-07

**Authors:** Alessandro Russo, Riccardo Serraino, Francesca Serapide, Andrea Bruni, Eugenio Garofalo, Federico Longhini, Enrico Maria Trecarichi, Carlo Torti

**Affiliations:** a Infectious and Tropical Disease Unit, Department of Medical and Surgical Sciences, ‘Magna Graecia’ University of Catanzaro, Italy; b Anesthesia and Intensive Care Unit, Department of Medical and Surgical Sciences, ‘Magna Graecia’ University of Catanzaro, Italy

**Keywords:** CAPA, COVID-19, Aspergillus, Galactomannan, Isavuconazole

## Abstract

Since 2020, cases of COVID-19-associated pulmonary aspergillosis (CAPA) have been frequently described, representing an important cause of mortality, especially among patients admitted to intensive care unit (ICU). A predisposition to invasive infection caused by *Aspergillus* spp. in SARS-CoV-2 infected patients can be ascribed either to the direct viral-mediated damage of the respiratory epithelium or to the dysregulated immunity associated with COVID-19.

In this case series we have collected the clinical, laboratory and radiological data of 10 patients admitted to the ICU with diagnosis of probable CAPA, according to the recent expert consensus statement, from March 2020 to December 2022 in the Teaching Hospital of Catanzaro in Italy. Overall, 249 patients were admitted to the COVID-19-ICU from March 2020 to December 2022; out of these, 4% developed a probable CAPA. Most of patients were male with a mean age of 62 years. Only two patients had an underlying immunocompromising condition. The observed mortality was 70%. In our institution, all COVID-19 patients requiring invasive mechanical ventilation systematically underwent bronchoscopy with bronchoalveolar lavage for an early evaluation of bacterial and/or fungal co- or super-infections, including galactomannan test. Patients were re-evaluated by an infectious diseases consultant team every 24–48 hours and the galactomannan test was systematically repeated based on patient's clinical course.

Even though the numbers in this study are very small, we report our experience about the role of early diagnosis and careful choice of antifungal therapy, considering the fragility of these patients, and its relationship with outcomes. Despite a systemic approach allowing early diagnosis and initiation of anti-fungal therapy, the mortality rate turned out to be very high (70%).

## 1 Introduction

Viral pneumonia increases patients’ susceptibility to bacterial and fungal superinfections, including invasive pulmonary aspergillosis (IPA). Influenza-associated pulmonary aspergillosis has been described in patients with influenza and severe acute respiratory distress syndrome (ARDS) in the intensive care unit (ICU) and is no longer considered a rare complication [ [1,2]]. It mainly affects patients with COVID-19 in critical care settings with ARDS, who require intensive care and mechanical ventilation. Respiratory viruses cause direct damage to the airway epithelium, enabling aspergillus to invade tissue. Furthermore, viral infection hampers ciliary clearance and leads to immune dysfunction or dysregulation, or both, locally or systemically [[3], [4], [5]]. The dysregulation of the immune system associated with ARDS is known, the mechanisms of which are still unclear, but some patients develop pronounced immunosuppression, which facilitates bacterial and fungal superinfection. In addition, steroid use has been identified as an important risk factor for both IPA susceptibility and mortality [6].

Since the early phase of the SARS-CoV-2 pandemic, cases of COVID-19-associated pulmonary aspergillosis (CAPA) in critically ill patients were described, and many studies were conducted to better outline this topic [7].

Different incidences of CAPA were worldwide registered. The reported incidence rate in critically ill patients ranged from 0 to 34.3%, with variability strongly impacted by the study period, being very low in the first COVID- 19 waves and gradually increasing over time. A recent consensus statement on defining and managing CAPA has been prepared by experts and supported by medical mycology societies [8]. However, diagnosis and classification remain challenging.

CAPA contributes substantially to a high mortality rate in COVID-19 patients of around 50% even though in several studies CAPA is the main cause of death from 17% to 30% [9].

During clinical practice in our hospital, of 249 patients admitted to the COVID-19-ICU from March 2020 to December 2022, we observed 10 cases of CAPA in ICU patients. This case series provides a description of all patients with CAPA admitted to the ICU in our hospital, highlighting the complexity and high mortality of this pathology and highlighting some aspects of therapeutic management that would require more clinical studies. The main aim of this case series is to describe a real-life experience providing the clinical characteristics and treatment outcome of patients.

## 2 Methods

This is a case series of COVID-19 patients admitted at “*Mater Domini*” Teaching Hospital of Catanzaro, Calabria Region, Italy in the Intensive Care Unit (ICU). The charts of patients admitted to the ICU from March 2020 to December 2022 in our Hospital with a probable diagnosis of CAPA, according to the recent expert consensus [8], were analyzed. Informed consent was obtained from the patient(s) (or relative/guardian) for the publication of all images, clinical data and other data included in the main manuscript.

The diagnosis of COVID-19 was made according to the WHO interim guidance. Specifically, nasopharyngeal swabs were tested by reverse transcriptase-polymerase chain reaction according to international standards. For each patient, several demographic data and clinical characteristics were recorded.

All patients tested positive due to worsening clinical condition and the onset of acute respiratory failure were transferred to the ICU of our hospital and treated with invasive mechanical ventilation following noninvasive mechanical ventilation.

**Fig. 1:**
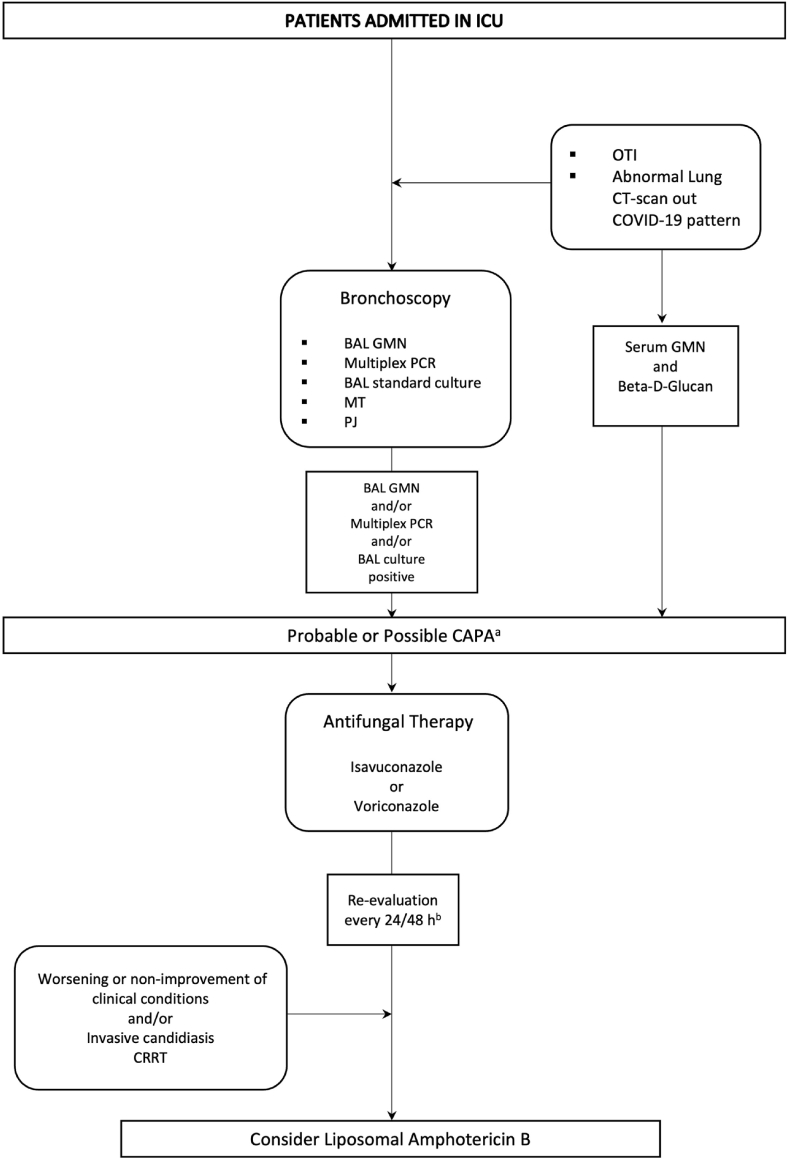
Management of ICU patients with COVID-19 currently admitted in our Institution. **Legend.** ICU: intensive care unit; GMN: galactomannan; OTI: oro-tracheal intubation; ARDS: acute respiratory distress syndrome; MT: *Mycobacterium tuberculosis*; PJ: *Pneumocystis jirovecii*; CRRT: continuous renal replacement therapy; ECMO: extracorporeal oxygenation.^a^ ECMM/ISHAM consensus definition of CAPA cases.^b^ Patients were re-evaluated by an infectious diseases consultant team every 24–48 hours.

All patients underwent high resolution computed tomography (HRCT) of the chest and a bronchoscopy with fluid collection from bronchoalveolar lavage (BAL) on which microbiological tests, including galactomannan (GMN) antigen and culture tests, were performed. Serum GMN detection was also performed. Detection of GMN on BAL and on serum was performed with immunoenzymatic test (BAL: ≥ 1.0 index; serum ≥ 0,50 index). Finally, the bacterial and fungal co- or super-infections of each patient determined by isolation from blood cultures or by BAL culture were also recorded. Patients were re-evaluated by an infectious diseases consultant team every 24–48 hours and the GMN test was repeated based on patient's clinical course (see Fig. 1).

## 3 Case series and discussion

Characteristics of patients with probable CAPA are reported in Table 1. Out of 249 patients admitted to the COVID-19-ICU from March 2020 to December 2022, 10 (4%) patients developed probable CAPA. Most patients were male (n = 7) and with a mean age of 62 years, in line with data in literature [10]. Only two patients (n° 6 and n° 10) showed an underlying immunocompromised condition. Three patients were discharged while the other 7 died. The observed mortality was 70%, despite an early diagnosis and an early targeted antifungal therapy.

**Table 1:** Patient's characteristics and outcome.

ICU										
No	Sex	Age in years	Medical History	BAL GMN	BAL Culture	Serum GMN	Radiological findings	Coinfections	**Antifungal treatment**	Outcome
1	M	60	No	Positive	A.niger	Negative	• Ground-glass opacity	A. baumannii (BAL- BC)	Isavuconazole 200 mg 3 times daily (six doses), 200 mg once daily	Died
							• Pulmonary consolidation			
**2**	M	58	COPD DM-II Obesity	Positive	A.niger	Negative	• Ground-glass opacity	No	Isavuconazole 200 mg 3 times daily (six doses), 200 mg once daily	Died
**3**	M	65	No	Positive	Negative	Negative	• Cavitations	A. baumannii	Isavuconazole 200 mg 3 times daily (six doses), 200 mg once daily	Died
							• Pulmonary consolidation	Pneumocystis jirovecii (BAL)		
**4**	M	58	No	Positive	Aspergillus spp	Negative	• Pulmonary consolidation	A.baumannii (BC – BAL)	Isavuconazole 200 mg 3 times daily (six doses), 200 mg once daily	Died
							• Ground-glass opacity			
**5**	F	73	No	Positive	A. flavus	Negative	• Pulmonary consolidation	A.baumannii (BAL)	Isavuconazole 200 mg 3 times daily (six doses), 200 mg once daily	Died
**6**	M	65	MM DM-II CKF	Positive	Negative	Positive	• Pulmonary consolidation	No	Isavuconazole 200 mg 3 times daily (six doses), 200 mg once daily	Discharged
							• Ground-glass opacity			
**7**	M	79	No	Positive	A. fumigatus	Negative	• Pulmonary consolidation	No	Isavuconazole 200 mg 3 times daily (six doses), 200 mg once daily	Died
							• Ground-glass opacity			
**8**	F	77	MS	Positive	Negative	Negative	• Pulmonary consolidation	C. parapsilosis	Isavuconazole 200 mg 3 times daily (six doses), 200 mg once daily	Died
							• Ground-glass opacity	P. aeruginossa K.pneumoniae (BC)	Then, Liposomal amphotericin B 5 mg/kg/day	
**9**	M	36	Oligophrenic	Positive	Negative	Negative	• Pulmonary consolidation	A. baumannii	Isavuconazole 200 mg 3 times daily (six doses), 200 mg once daily	Discharged
								C.albicans (BC)	Then, Liposomal amphotericin B 5 mg/kg/day	
**10**	F	50	Heart-lung transplantation, Obesity	Positive	A.fumigatus	Negative	• Pulmonary consolidation	No	200 mg 3 times daily (six doses), 200 mg once daily	Discharged

**Legend.** BAL: bronchoalveolar lavage; GMN: galactomannan; BC: blood cultures; COPD: chronic obstructive pulmonary disease; DM: diabetes mellitus; MM: multiple myeloma; MS: multiple sclerosis.

Here we report 3 emblematic cases observed in our COVID-19-ICU in the study period that highlight the challenges in the therapeutic management of this disease, especially in ICU patients.

Case 2 in Table 1.

A 57-years-old patient with a history of diabetes mellitus type 2 and chronic obstructive pulmonary disease was admitted at day 0 (D 0) in ICU in critical condition and ARDS and immediately underwent orotracheal intubation (OTI). At chest HRCT diffuse areas of ground glass opacity was found. The patient was treated for COVID-19 with standard of care without improvement of clinical condition. Bronchoscopy was performed on the day after admission (D 1) with BAL-positive GMN finding and isavuconazole was started (D 2). Because of worsening pulmonary gas exchange, the patient was treated with extracorporeal membrane oxygenation (ECMO) (D 2). Culture test on BAL was positive for Aspergillus niger and serum GMN was negative, no other isolates of pathogens. In the second week after admission the patient died.

Case 3 in Table 1.

**Fig. 2:**
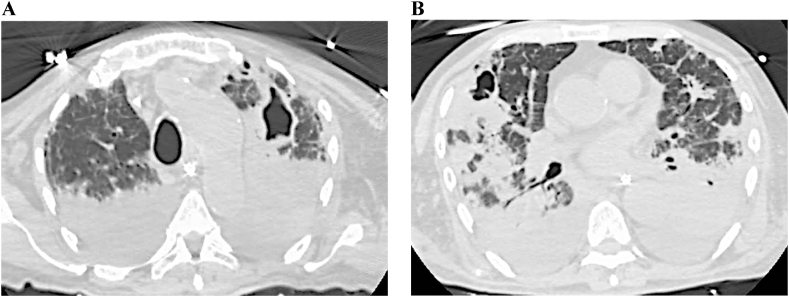
Case 3 chest HRCT. A B In the two HRTC scans (A and B) of this patient, extensive areas of lung consolidation and concomitant bilaterally excavated formations are visible.

A 65-years-old patient was admitted in critical condition and ARDS and underwent immediately OTI. At chest HRCT, massive areas of pulmonary consolidation and concomitant excavated formations bilaterally were found (see Fig. 2) (D 0). The patient was treated for COVID-19 with standard of care without improvement of clinical condition. Bronchoscopy was performed on the day after admission (D 1) with BAL-positive GMN finding and isavuconazole was started (D 3). Culture test on BAL was positive for Acinetobacter baumanni and serum GMN was negative. Molecular test on BAL was positive for Pneumocystis jirovecii and targeted antibiotic therapy was initiated was started (D 4). In the third week after admission the patient died.

Case 8 in Table 1.

A 77-year-old unvaccinated against SARS-CoV-2 patient with a history of multiple sclerosis. The patient was admitted in critical clinical condition and ARDS and immediately underwent OTI. At chest HRCT a ground glass opacity pattern and pulmonary consolidations were found (D 0). The patient was treated for COVID-19 with the standard of care without improvement of general clinic condition. On the fifth day from admission bronchoscopy was performed (D 5) with a BAL-positive GMN finding, and isavuconazole was started (D 6). Culture test on BAL was negative and GMN serum was negative. In the fourth week of treatment, blood cultures were positive for *Candida parapsilosis*. Multiple positive blood cultures for different bacterial pathogens were found during the hospitalization and antibiotic therapy was undertaken. The patient died after three months from the admission.

In our experience we fixed the four different waves as follows: the first from March 23, 2020 to April 30, 2020; the second one from October 11, 2020 to February 25, 2021; the third from March 09, 2021 to September 25, 2021; the fourth from November 02, 2021 to June 23, 2022. In this case series we found that patients with probable CAPA diagnoses in our hospital occurred from the third wave, from March 2021 to December 2022. This finding could be based on three main reasons: during the first two waves the use of bronchoscopy and, thus, BAL analysis was significantly reduced due to its nature of aerosol generation and high risk of viral transmission [11]. Furthermore, during the second wave, corticosteroid therapy was introduced and approved in the hospital management of COVID-19 patients [12] and corticosteroid therapy was associated with a significant increase in the risk of CAPA [13]. Finally, GMN test on BAL fluid was systematically detected from the second wave of pandemic. Consequently, no definitive data can be reported in this study about the incidence of CAPA in the different waves of pandemic.

The definition of case of CAPA is complex and controversial. The European Organization for Research and Treatment of Cancer (EORTC)/Mycosis Study Group Education and Research Consortium (MSGERC) case definition of invasive fungal disease is rarely applicable because it only applies to patients with host-specific factors, which are typically absent in patients suspected of having CAPA [14]. In fact, most patients with CAPA do not have an underlying immunocompromising condition. For this reason, in the AspICU study *Blot* et al. recently developed disease definitions aimed at ICU populations. These criteria required a positive culture for *Aspergillus* as an entry criterion, although cultures are negative in most cases, and were later revised by *Schauwvlieghe* et al. [15,16]. In December 2020, the European Confederation of Medical Mycology (ECMM) and the International Society of Human and Animal Mycology (ISHAM) published a consensus definition of CAPA cases [8], classifying patients into proven, probable, and possible CAPA. Bronchoscopy with BAL remains the cornerstone of CAPA diagnosis. The diagnostic yield of serum GMN is low in CAPA as, at best, 20% of patients showed positive results, and proven CAPA cases have been reported with negative serum GMN [17,18].

In the absence of uniform diagnostic criteria, a wide range of mycological criteria was used in the first major studies to classify patients with CAPA. To avoid aerosol exposure and transmission, there was also a reluctance to perform bronchoscopies, and many of these studies relied on nonspecific mycologic tests performed on the tracheal aspirate [19] or the beta-D-glucan test from serum, which is rather a general marker of fungal infection in the ICU than specific for CAPA [20]. These circumstances, combined with the nonspecific clinical and radiological presentation of CAPA in patients with COVID-19, have probably led to overestimation of cases and high variability. After 2020, the application of the more stringent ECCM/ISHAM criteria led to a significant reduction in the CAPA prevalence rate [21].

In our case series, the systematic approach for an early diagnosis of bacterial and/or fungal co- and super-infections with bronchoscopy and microbiological testing on BAL, including GMN, in all patients undergoing invasive mechanical ventilation, allowed an early detection of probable CAPA cases with a subsequent early start of a targeted antifungal therapy.

About radiological findings, we found at chest HRCT 6 patients with the typical COVID-19 aspect, including peripheral bilateral ground-glass opacities with or without consolidation in the early phase; multifocal ground-glass opacities with or without consolidation or crazy paving in the peak phase. Cavitated lesions were present at HRCT of only 1 patient. However, it is now known that COVID-19 pneumonia may present with radiological features like those of CAPA, and vice versa, so typical radiological signs of CAPA may go unrecognized. For an appropriate diagnosis of CAPA, it will be necessary to consider that many cases of CAPA may occur without clear radiological evidence and overlap with CT scans with typical or indeterminate appearance for COVID-19 [8,22,23].

Our patients were all treated with isavuconazole (loading dose of 200 mg three times daily for six doses, followed by 200 mg once daily, 12–24 hours after the last loading dose). We found that 6 patients showed a concomitant bacterial co-infection that required antibiotic treatment. Of interest, candidemia due to *C. albicans* and *C. parapsilosis* was found in two patients, respectively, during treatment with isavuconazole. In general, voriconazole and isavuconazole are the recommended first-line treatment for invasive pulmonary aspergillosis [24,25], while echinocandins are not recommended for use as monotherapy in IPA; However, when combined with an azole, they could lead to a therapeutic advantage in critically ill patients. Furthermore, in areas where there is a high prevalence of azole-resistant *Aspergillus* spp, combination therapy may extend antifungal coverage until minimal inhibitory concentrations become available [[26], [27], [28], [29]]. The optimal duration of therapy is unknown; the expert panel suggests a treatment course of 6–12 weeks. Although radiological imaging of the lung may not be a useful indicator, a follow-up lung CT scan to document the resolution of pulmonary infiltrates before ending treatment would seem reasonable [8].

Of importance, in our case series, although patients were treated early with isavuconazole, mortality rate remained very high (70%) about 20 points higher than in other cohorts. This could be due to the very severe clinical condition of the patients and the presence of bacterial co-infections. However, in our study CAPA was attributed as the main cause of death in only two cases (20 %) in line with other patient cohorts [9]. There are many considerations about the high mortality rate in patients with CAPA.

Firstly, very serious clinical condition of patients and/or bacterial co- and super-infections can explain the excess of mortality in this population. Out of these some considerations can be also reported about isavuconazole [30]. Many factors, particularly common in patients admitted to the ICU with COVID-19, can influence the pharmacokinetics of isavuconazole. First of all, this might be influenced by body mass index (BMI), so patients with very high BMI might achieve lower therapeutic levels with standard dosing, while patients with low BMI might have higher exposure. This topic is very relevant as obese patients are frequently reported as subjects with CAPA. Therefore, treatment options against CAPA should consider the impact of BMI on their effectiveness [31].

In addition, low blood levels have been observed with ECMO, and perhaps continuous renal replacement therapy (CRRT). ICU patients may often require supportive therapies for renal or pulmonary failure, such as CRRT and ECMO, which may result in subtherapeutic levels of the drug. In these cases, therapeutic drug monitoring (TDM) of isavuconazole should be performed to proceed safely with antifungal treatment with isavuconazole, especially in patients when clinical monitoring is complicated by concomitant lung infections [32,33].

Finally, isavuconazole has not demonstrated efficacy on candidemia and invasive candidiasis, failing to demonstrate non-inferiority to caspofungin [34,35]. In our case series, 2 patients developed breakthrough candidemia during treatment with isavuconazole. Many hypotheses could explain this phenomenon, including that isavuconazole can show, as fluconazole, trailing effect in which a total inhibition of fungal growth is not achieved (also using increasing concentrations of antifungal drugs), and the strains maintain a residual growth at high antifungal concentrations [36].

In these situations, liposomal amphotericin B can be considered an important choice to treat these patients despite the increased risk of side effects, especially when TDM is not feasible. In geographical area with high levels of azole resistance, it was recommended to cover resistance in initial antifungal therapy by treating with liposomal amphotericin B. When azole resistance is detected liposomal amphotericin B is recommended [8,37,38]. However, liposomal amphotericin B may be considered also for the antifungal treatment of CAPA patients if the determination of azole levels is not possible or if there is a high risk of drug interactions. Finally, superinfection by *Candida* spp can require the use of a drug, like liposomal amphotericin B, that show a spectrum of activity against *Candida* spp and *Aspergillus* spp.

The limitation of this study is the small number of CAPA cases analyzed furthermore, because of the retrospective nature of the study, we could not retrieve the necessary clinical and diagnostic details of all patients and did not allow us a more detailed analysis of the cases.

## 4 Conclusions

In this case series, despite the small numbers, it emerges that CAPA represents a fearsome and frequent condition with a high mortality. In our experience, early diagnosis by bronchoscopy and initiation of appropriate antifungal therapy is of key importance in patients with COVID-19 severe respiratory failure as well as close monitoring. Despite this approach, mortality among patients admitted to ICU with CAPA remains very high. This may be due to a very serious clinical condition of the patients or to the presence of other concomitant bacterial co-infections. However, a careful choice of antifungal therapy is very important considering all the characteristics of critically ill patients admitted in ICU for COVID-19. In particular, the use of liposomal amphotericin B in the treatment of CAPA could be more frequently considered, especially in patients undergoing ECMO or CRRT. Data form these case series tried to summarize the most important issues about characteristics of patients, diagnosis, management and therapy of CAPA. For this reason, clinical trials should be conducted in this direction.

## CRediT authorship contribution statement

**Alessandro Russo:** Writing - review & editing, Writing - original draft, Supervision, Data curation, Conceptualization. **Riccardo Serraino:** Writing - review & editing, Writing - original draft, Data curation. **Francesca Serapide:** Writing - original draft, Data curation. **Andrea Bruni:** Writing - original draft, Supervision. **Eugenio Garofalo:** Writing - original draft, Supervision. **Federico Longhini:** Writing – original draft, Supervision. **Enrico Maria Trecarichi:** Writing – review & editing, Writing – original draft, Supervision. **Carlo Torti:** Writing – original draft, Supervision, Conceptualization.

## Declaration of competing interest

The authors declare that they have no known competing financial interests or personal relationships that could have appeared to influence the work reported in this paper.
